# Identification of a novel *ANK1* gene variant c.1504-9G>A and its mechanism of intron retention in hereditary spherocytosis

**DOI:** 10.3389/fgene.2024.1390924

**Published:** 2024-04-09

**Authors:** Ting Xiong, Zhongjin Xu, Qian Wan, Feng Chen, Yao Ye, Hong Wang, Chongjun Wu

**Affiliations:** ^1^ Department of Endocrine Genetics and Metabolism, Jiangxi Provincial Children’s Hospital, Nanchang, China; ^2^ Department of Hematology, Jiangxi Provincial Children’s Hospital, Nanchang, China

**Keywords:** ANK1, hereditary spherocytosis, intron retention, whole-exome sequencing, minigene splicing assay

## Abstract

**Objective:** The objective of this study was to pinpoint pathogenic genes and assess the mutagenic pathogenicity in two pediatric patients with hereditary spherocytosis.

**Methods:** We utilized whole-exome sequencing (WES) for individual analysis (case 1) and family-based trio analysis (case 2). The significance of the intronic mutation was validated through a Minigene splicing assay and supported by subsequent *in vitro* experiments.

**Results:** Both probands received a diagnosis of hereditary spherocytosis. WES identified a novel *ANK1* c.1504-9G>A mutation in both patients, causing the retention of seven nucleotides at the 5′ end of intron 13, as substantiated by the Minigene assay. This variant results in a premature stop codon and the production of a truncated protein. *In vitro* studies indicated a reduced expression of the *ANK1* gene.

**Conclusion:** The novel *ANK1* c.1504-9G>A variant is established as the causative factor for hereditary spherocytosis, with the c.1504-9G site functioning as a splicing receptor.

## 1 Introduction

Hereditary spherocytosis (HS) or spherocytosis type 1 (MIM: # 182900) is an inherited hemolytic disorder commonly characterized by symptoms of extravascular hemolysis, including anemia, jaundice, and splenomegaly. HS has a global prevalence, with incidences reported as high as 1 in 2,000 in European and North American populations (Bolton-Maggs et al., 2012). In China, a comprehensive review of literature from 1978 to 2013 by Wang et al. (2015) estimated the overall prevalence of HS at approximately 1.37 per 100,000, with a slight gender discrepancy of 1.27 cases per 100,000 in males and 1.49 cases per 100,000 in females, indicating that HS is the most prevalent Mendelian red cell membrane disorder in the country (Tao et al., 2016). Genetic mutations in the *ANK1*, *SPTB*, *SPTA1*, *SLC4A1*, and *EPB42* genes lead to defects in the corresponding ankyrin, β-spectrin, α-spectrin, band 3, and protein 4.2, respectively. These defects result in a decreased erythrocyte membrane surface area, increased osmotic fragility, and ultimately, the transformation of red blood cells from their typical biconcave shape to a spherical morphology. This morphological change predisposes the red blood cells to hemolysis within the spleen (Mohandas and Gallagher, 2008). Currently, China lacks a disease registry system for HS, and there is a significant need for epidemiological data. Although the incidence and detection rates of HS have been on the rise in recent years, misdiagnosis and oversight of the condition remain prevalent (Chen et al., 2020; Gerard et al., 2020; Zhu et al., 2020). In this context, we present two cases of HS attributed to the same novel *ANK1* intronic mutation, which we have demonstrated to function as a splicing receptor.

## 2 Materials and methods

### 2.1 Objects

Upon admission, the two unrelated probands displayed varying degrees of anemia. Comprehensive baseline laboratory evaluations were conducted, including assessments of ferritin, iron levels, transferrin, total iron binding capacity, folate, and vitamin B12, glucose-6-phosphate dehydrogenase (G-6-PD) activity, hemoglobin electrophoresis, direct antiglobulin test, thalassemia gene screening, and bone marrow analysis, in accordance with the locally prevalent anemia etiologies. Both cases were thoroughly investigated to rule out common anemia causes such as nutritional deficits, G-6-PD enzyme deficiency, thalassemia, autoimmune hemolytic anemia, and bone marrow hematopoietic disorders.

### 2.2 Methods

#### 2.2.1 Sample collection

Following informed consent from the family, 4 mL of EDTA-anticoagulated peripheral venous blood from each child and 2 mL from each parent were collected. These samples were then forwarded to Chigene (Beijing) Translational Medical Research Center Co. Ltd. (Beijing, China) for trio whole-exome sequencing (trio-WES) and subsequent bioinformatic analysis.

#### 2.2.2 Whole-exome sequencing (WES)

WES was performed using the xGen Exome Research Panel v2.0 (Integrated DNA Technologies, United States) to construct an exome library. High-throughput sequencing was carried out on the NovaSeq 6000 platform (Illumina, United States). The sequencing process, including data generation, cleaning, and quality control, adhered to the manufacturer’s recommended protocols, achieving an average sequencing depth of 100X and an exomic coverage of no less than 99%. WES data were subjected to automated bioinformatics analysis through the Chigene Comprehensive Genetic Disease Precision Diagnosis Cloud Platform (https://www.chigene.cn/zaixianfenxipingtai/). This analysis generated insertions/deletions (indels) and single nucleotide variant data of ≤50 bp and flagged copy number variations spanning multiple consecutive exons using proprietary algorithms developed by Chigene. The variant database integrated into the Chigene Cloud Platform, including resources such as dbSNP, ClinVar, HGMD pro, gnomAD, and OMIM, provided annotations for the detected gene variants, including minor allele frequency (MAF), reported pathogenicity cases, literature, and associated diseases of the variant genes. The pathogenicity of gene variants was classified according to the clinical practice guidelines of the American College of Medical Genetics and Genomics (ACMG), and categorized as pathogenic, likely pathogenic, of uncertain significance, likely benign, or benign.

#### 2.2.3 Pathogenic variant confirmation

Sanger sequencing was conducted utilizing the ABI3730 (Thermo Fisher Scientific, Waltham, United States) sequencer, adhering to the manufacturer’s protocols. The reference for the *ANK1* DNA sequence was NCBI transcript version NM_001142446.

#### 2.2.4 *In vitro* analysis of *ANK1* c.1504-9G>A

##### 2.2.4.1 Minigene tests the effect of mutations on gene splicing

Minigene fishing techniques were employed to construct the recombinant vectors pcMINI-wt/mut and pcDNA3.1-wt/mut, incorporating restriction sites. These vectors were then transiently transfected into Hela and 293T cell lines. Total RNA was extracted from the cell samples, and PCR amplification was performed using primers flanking the minigene. The transcriptional band size was evaluated by agarose gel electrophoresis and confirmed by sequencing.

##### 2.2.4.2 Functional analysis

###### 2.2.4.2.1 Vector engineering

The p3Xflag-CMV-7.1-wt vector was engineered using the synthesized whole gene *ANK1* CDS as a template, with p3Xflag-CMV-7.1-*ANK1*-EcoRI-F and p3Xflag-CMV-7.1-*ANK1*-KpnI-R as primers. Similarly, the p3Xflag-CMV-7.1-mut vector was constructed using p3Xflag-CMV-7.1-*ANK1*-EcoRI-F and CMV-7.1-*ANK1*-AfeI-R primers. The integrity of the constructed vectors was confirmed by sequencing.

###### 2.2.4.2.2 Cell culture and gene delivery

293T cells were propagated in DMEM supplemented with 10% fetal bovine serum. The cells were then transiently transfected with the constructed wild-type and mutant eukaryotic expression vectors using Lipo2000 reagent, as per the manufacturer’s instructions. After 48 h post-transfection, samples were harvested for quantitative PCR (qPCR) and Western blot (WB) analyses.

###### 2.2.4.2.3 mRNA expression analysis

Cell samples were collected 48 h post-transfection with the recombinant expression vectors. Total RNA was isolated using the Trizol method, followed by DNA digestion and cDNA synthesis. The qPCR technique was utilized to quantify the expression levels of the wild-type and mutant genes.

###### 2.2.4.2.4 Protein expression assessment

Cellular precipitates were obtained 48 h after transfection with the expression vectors. Total cellular protein was extracted using RIPA buffer, and protein concentrations were determined using a BSA assay kit. Subsequent to protein denaturation, Western blot analysis was performed to compare the expression levels of the wild-type and mutant proteins.

## 3 Results

### 3.1 Clinical characteristics

The comprehensive clinical features of the probands are delineated in Supplementary Material S1 [including a case previously documented by our group (Wu et al., 2021)]. Both unrelated patients were of Han Chinese descent and exhibited varying degrees of anemia upon admission. All diagnostic evaluations were conducted prior to any splenectomy or blood transfusion procedures.

### 3.2 Discovery of *ANK1* c.1504-9G>A variant

**FIGURE 1:**
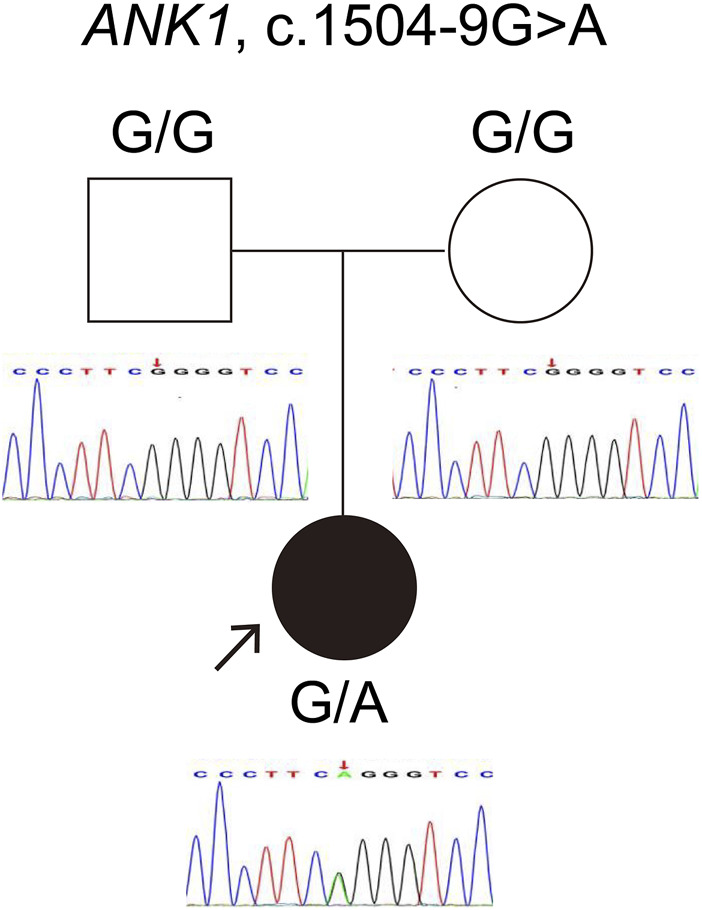
Verification of the *ANK1* gene variant c.1504-9G>A in Case 2. The red arrow highlights the mutation site confirmed by Sanger sequencing.

The *ANK1* c.1504-9G>A variant was identified in both individuals, a finding first reported by our team (Wu et al., 2021). For case 1, genetic co-segregation analysis was not feasible due to the unavailability of parental samples. In case 2, the mutation emerged *de novo* and was classified as likely pathogenic according to ACMG standards. Sanger sequencing confirmation for case 2 is depicted in Figure 1.

### 3.3 *In vitro* assessment of *ANK1* c.1504-9G>A

#### 3.3.1 *ANK1* c.1504-9G>A induces retention of 7 nucleotides at the 5′ end of intron 13

**FIGURE 2:**
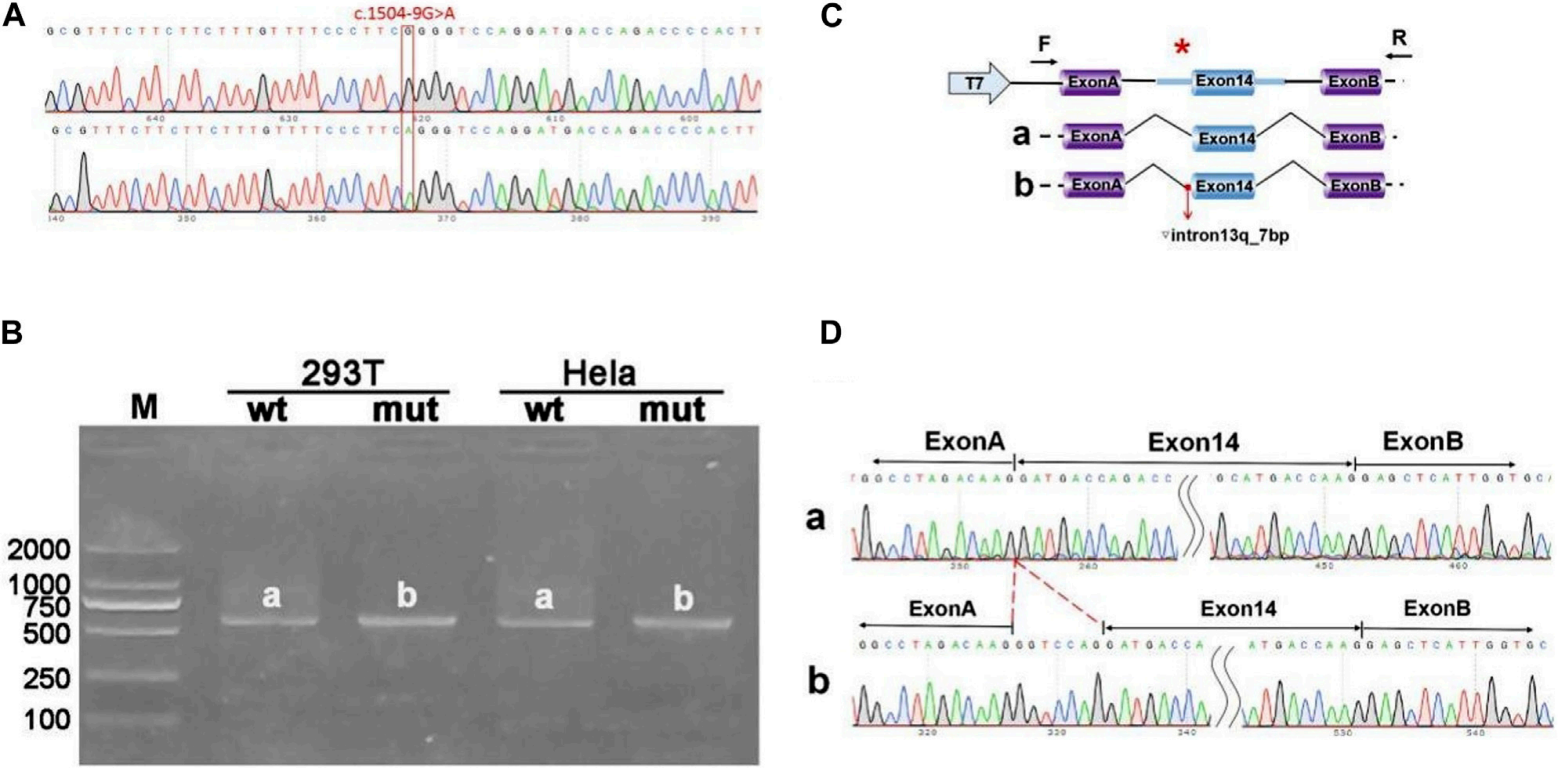
Results from the pcMINI constructs. **(A)** Sequencing chromatograms of minigene constructs, with the wild type (WT) on top and the mutant (MUT) below. **(B)** Agarose gel electrophoresis of RT-PCR products for transcript analysis. **(C)** Illustration of the minigene construction strategy and expected splicing products, with bands observed in Hela and 293T cells denoted as a and b, respectively. **(D)** The sequencing results corresponding to the spliced products. Red * indicates mutation location.

The pcMINI-*ANK1*-wt/mut minigene construct was designed to incorporate a segment of intron 13 (397bp), exon 14 (198bp), and part of intron 14 (207bp) into the pcMINI vector. Post-transfection analysis revealed a spliced sequence spanning ANK1 exon A through exon 14 to exon B. RT-PCR results indicated the presence of a band of the predicted size (587bp), termed band a, in the wild type, and an additional band, referred to as band b, in the mutant type. Sequencing of bands a and b confirmed band a as a normally spliced sequence, following the pattern exon A-exon 14 (198nt)-exon B. Conversely, band b exhibited a 7 nucleotide retention on the right side of intron 13, with a splicing pattern of exon A-▽ intron 13 (7nt)-exon 14 (198nt)-exon B (Figure 2).

#### 3.3.2 Analysis of pcDNA3.1-*ANK1*-wt/mut constructs

**FIGURE 3:**
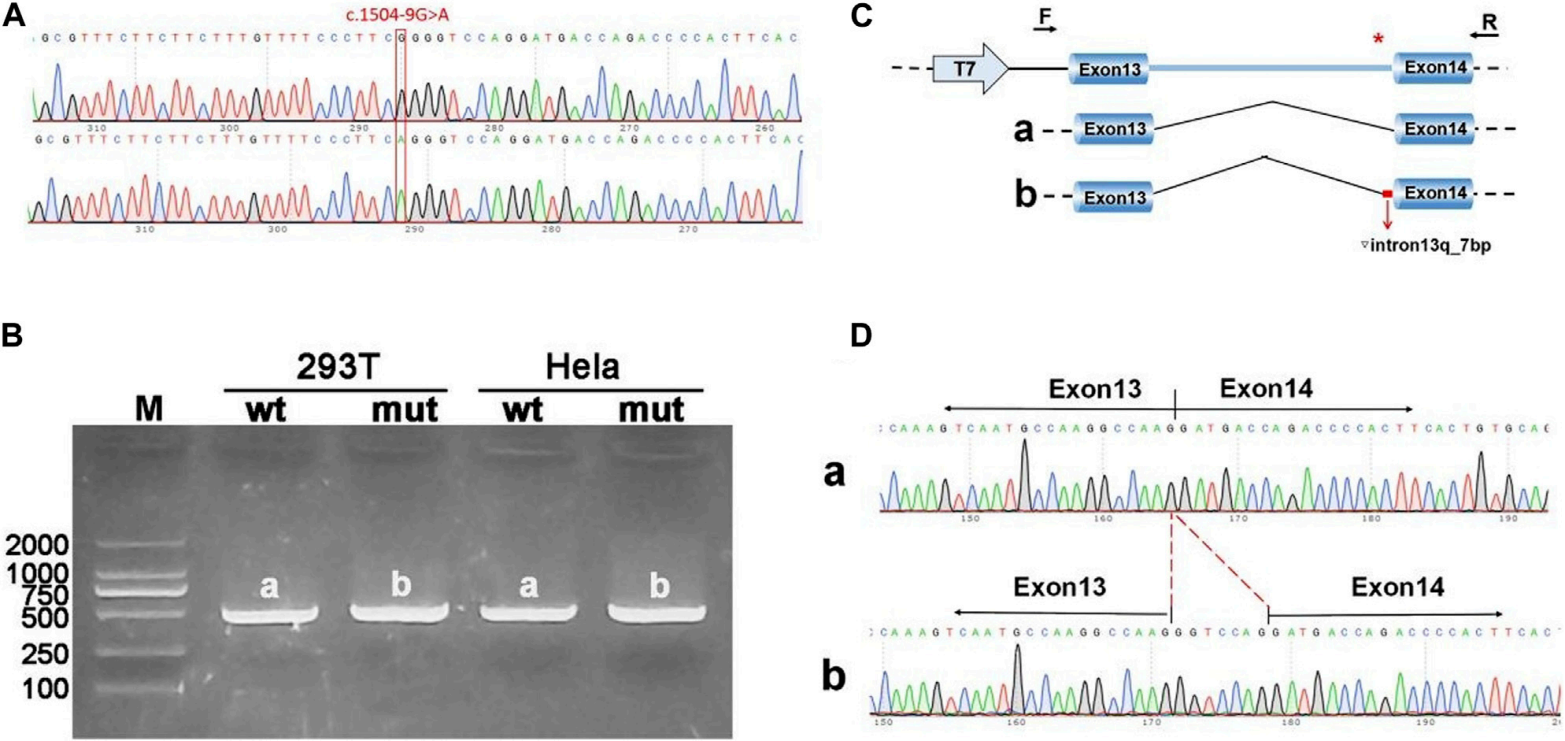
Results from the pcDNA3.1 constructs. **(A)** Sequencing chromatograms of minigene constructs, with the wild type (WT) on top and the mutant (MUT) below. **(B)** Agarose gel electrophoresis of RT-PCR products for transcript analysis, with bands in Hela and 293T cells labeled as a and b, respectively. **(C)** Schematic representation of the minigene construction strategy and expected splicing patterns. **(D)** The sequencing results corresponding to the spliced products. Red * indicates mutation location.

The pcDNA3.1-*ANK1*-wt/mut minigene strategy involved inserting a fragment encompassing exon 13 (99bp), intron 13 (1103bp), and exon 14 (198bp) into the pcDNA3.1 vector. Transfection was followed by observation of the exon 13-exon 14 splicing pattern for abnormalities. RT-PCR findings revealed a band corresponding to the expected size (507bp), designated as band a in the wild type, and a mutant-specific band b. Sequencing of these bands showed that band a represented a normal splicing sequence, exon 13 (99nt)-exon 14 (198nt). Band b, however, retained an additional 7 nucleotides at the right side of intron 13, with a splicing sequence of exon 13 (99nt)-▽ intron 13 (7nt)-exon 14 (198nt) (Figure 3).

#### 3.3.3 Functional analysis of the *ANK1* c.1504-9G>A variant

**FIGURE 4:**
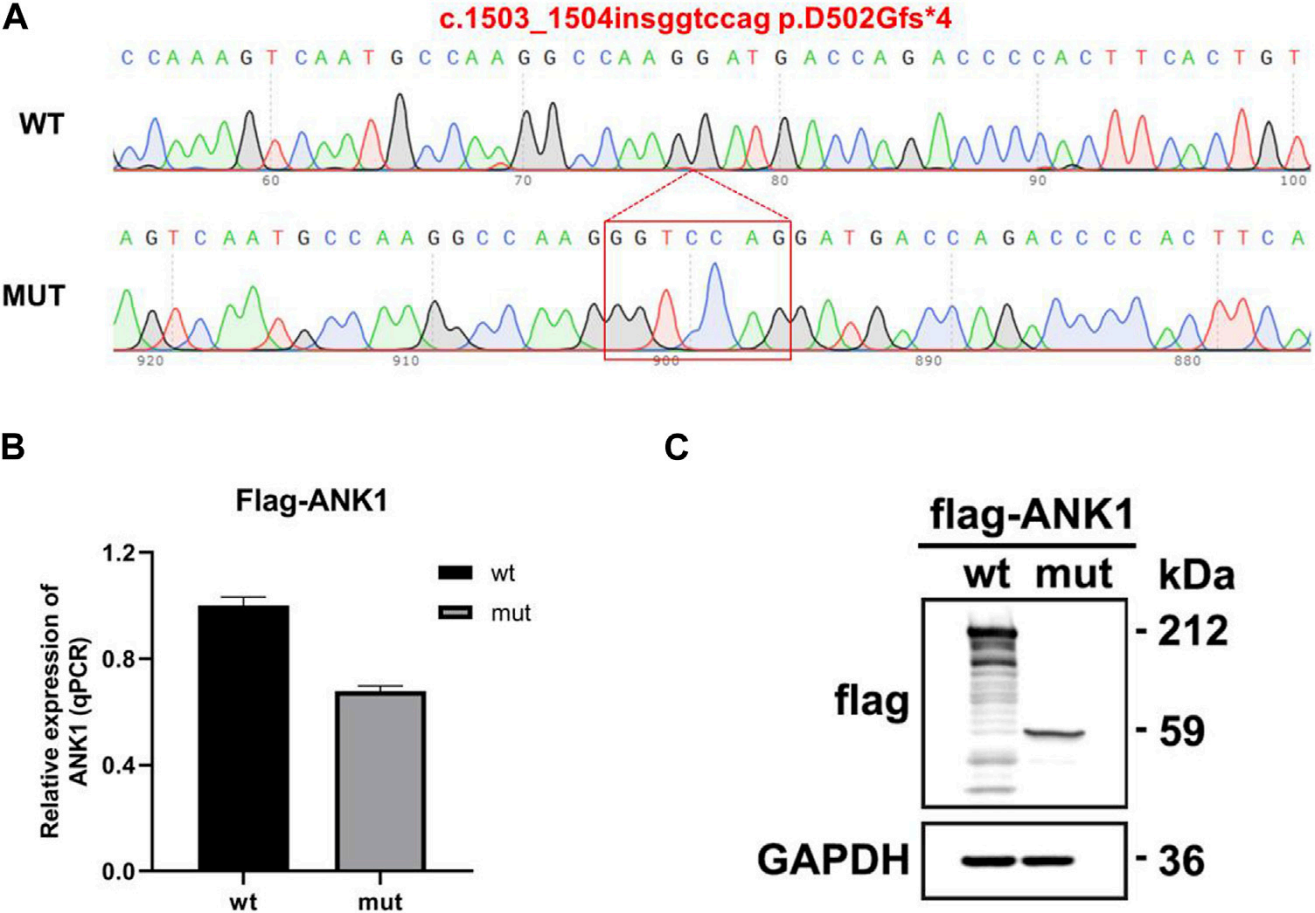
Gene expression analysis using the p3Xflag-CMV-7.1 vector. **(A)** Sequencing confirmation of the successful construction of the mutant variant: c.1503_1504insggtccag p.D502Gfs*4. **(B)** Quantitative PCR (qPCR) detection of mRNA expression levels. **(C)** Western blot (WB) analysis for the assessment of protein expression.

The eukaryotic expression vectors p3Xflag-CMV-7.1-*ANK1*-wt/mut were transfected into 293T cells, which were subsequently harvested after 48 h for analysis. Quantitative PCR (qPCR) was utilized to measure the expression levels of *ANK1* in the cells, with primers *ANK1*-3xFLAG-QPCR-F and -R specifically designed for the wild-type and mutant genes. The qPCR data indicated that the expression of the mutant *ANK1* gene was reduced to 67% of that observed in the wild-type. Western blot analysis confirmed the presence of proteins at the anticipated molecular weights: 212 kDa for the wild-type construct and 59 kDa for the mutant, indicating the synthesis of a truncated protein. These findings are illustrated in Figure 4.

## 4 Discussion

In this study, both patients exhibited the classic hereditary spherocytosis (HS), as known as spherocytosis type 1 (MIM: # 182900), phenotypes, as outlined in Supplementary Material S1. The etiology of HS is linked to defects in erythrocyte membrane proteins, which lead to a diminished surface area of red blood cells, altered sphericity, increased membrane fragility, and compromised elasticity and stability, all of which are associated with genetic mutations (Manciu et al., 2017). Deficiencies or dysfunctions in these membrane proteins disrupt the vertical connectivity of the membrane’s bilayer skeleton. HS is predominantly caused by mutations in the *ANK1*, *SPTB*, *SPTA1*, *SLC4A1*, and *EPB42* genes, which impact the integrity of ankyrin, β-spectrin, α-spectrin, band 3, and protein 4.2, respectively. Consequently, erythrocytes assume an abnormal spherical shape that predisposes them to hemolysis (Mohandas and Gallagher, 2008).

HS is characterized by a wide range of phenotypic and genotypic variability, with distinct prevalence rates and molecular patterns across various ethnicities and geographic regions. A review by Wang et al. (2021) of the global literature on HS in Chinese patients from 2000 to 2020, which included genetic and clinical data, revealed that *ANK1* (46%) and *SPTB* (42%) mutations are the most common genetic causes of HS, followed by *SLC4A1* (11%) and *SPTA1* (1%). Notably, no *EPB42* mutations were reported in Han Chinese individuals. Among *ANK1* defects, most mutations previously identified were frameshift, nonsense, or located at canonical splicing sites (defined as the two nucleotides at both the 5′ donor and 3′ acceptor splice sites), with mutations near splice sites being relatively rare (Aggarwal et al., 2020; Qin et al., 2020; Tole et al., 2020; Wang et al., 2018; Wang et al., 2020; Xie et al., 2021). The discovery of the novel c.1504-9G>A mutation in two unrelated HS patients within this study underscores its pathogenic significance.

The *ANK1* gene, comprised of 42 exons and located on chromosome 8p11.2, encodes the ankyrin protein, which consists of 1881 amino acids including the N-terminal 89 kD domain, the central 62 kD domain, and a variable C-terminal regulatory region (Park et al., 2016). Ankyrin plays a pivotal role in membrane stability by anchoring the β-spectrin tail at one end and the band 3 protein at the other, thereby securing the membrane skeleton within the lipid bilayer (Ipsaro et al., 2009). Alterations in the quantity or quality of ankyrin can undermine the connection between the membrane skeleton and the lipid bilayer, causing instability in the lipid bilayer, vesicle formation and lipid loss, reduction in erythrocyte membrane surface area, red blood cell sphericity (Barcellini et al., 2011), and ultimately hemolysis in the spleen.

In our research, we implemented two distinct molecular systems to validate the impact of intronic mutations on gene splicing, providing dual confirmation of our findings. The pcMINI-wt/mut system was utilized to monitor intron retention and exon skipping phenomena, whereas the pcDNA3.1-wt/mut system was primarily focused on detecting intron retention. The results from both systems consistently demonstrated that the *ANK1* c.1504-9G>A mutation leads to the retention of 7 nucleotides downstream of intron 13 during the splicing process. Consequently, we speculated that this retention of 7 nucleotides may result in either RNA degradation or the insertion of seven extraneous codons between exons 13 and 14 in the mature mRNA. It is noteworthy that frameshift mutations occurring between exons 13 and 14 have been known to induce premature translation termination, thereby producing a truncated *ANK1* protein.

The *in vitro* experiments conducted as part of this study have provided further insights into the cellular consequences of this particular variant. Our observations revealed that the analogous variant, c.1503_1504insggtccag p.D502Gfs*4, culminates in the generation of a prematurely truncated protein that terminates within exon 14, rather than being subjected to degradation. This truncated protein may potentially possess undefined biological functions. Additionally, the observed downregulation of *ANK1* mRNA expression underscores a deficiency in the production of the wild-type *ANK1* protein, corroborating the pathogenic nature of the mutation.

## 5 Conclusion

In summary, our study uncovered a novel *ANK1* c.1504-9G>A variant and established that it leads to the production of a truncated *ANK1* protein. Identifying this intronic mutation in proximity to the canonical splicing sites of the *ANK1* gene enhances our comprehension of the genotype-phenotype correlations in *ANK1*-associated hereditary spherocytosis. These findings pave the way for future research into the regulatory mechanisms of *ANK1* expression.

## Data Availability

The original contributions presented in the study are publicly available. This data can be found here: https://databases.lovd.nl/shared/variants/0000971537#00025918.
