# Correction: Identification of a novel *ANK1* gene variant c.1504-9G>A and its mechanism of intron retention in hereditary spherocytosis

**DOI:** 10.3389/fgene.2025.1762322

**Published:** 2026-01-02

**Authors:** Ting Xiong, Zhongjin Xu, Qian Wan, Feng Chen, Yao Ye, Hong Wang, Chongjun Wu

**Affiliations:** 1 Department of Endocrine Genetics and Metabolism, Jiangxi Provincial Children’s Hospital, Nanchang, China; 2 Department of Hematology, Jiangxi Provincial Children’s Hospital, Nanchang, China

**Keywords:** ANK1, hereditary spherocytosis, intron retention, whole-exome sequencing, minigene splicing assay

In the originally published article, the *ANK1* gene variant was incorrectly reported using transcript NM_020475.2 in the **Section of 2.2.3** Pathogenic variant confirmation. The correct reference transcript should be NM_001142446.

A correction has been made to the section Materials and methods, **subsection 2.2.3** Pathogenic variant confirmation:

“The reference for the *ANK1* DNA sequence was NCBI transcript version NM_001142446”.

The original article has been updated.

