# Supplementary material for: Identification of a novel ANK1 gene variant c.1504-9G>A and its mechanism of intron retention in hereditary spherocytosis
Source: Front Genet. 2024 Apr 9;15:1390924. doi: 10.3389/fgene.2024.1390924 (PMC11035775; doi:10.3389/fgene.2024.1390924)
Supplement: Supplementary file 1 [file Table1.DOCX]

**Supplementary material 1** Clinical characteristics of the probands.

| Case | 1(Wu et al.,2021) | 2 |
| --- | --- | --- |
| Sex | male | female |
| Age | Four months and 18 days | Nine months and 30 days |
| Chief Complaint | Experiencing pale yellow discoloration for 2 months. | Experiencing pale yellow discoloration for more than 9 months |
| WBC | 16.14 | 6.15 |
| RBC | 1.93 | 2.98 |
| HB | 55 | 66 |
| PLT | 335 | 529 |
| HCT | 18 | 19.7 |
| MCV | 93.3 | 82.8 |
| MCH | 28.5 | 27.7 |
| MCHC | 306 | 335 |
| RDW-SD | 83.3 | 46.1 |
| RDW-CV | 28.9 | 16.5 |
| RET# | 272.2 | 170.64 |
| RET | 14.13 | 7.11 |
| TBIL | 44.4 | 56.3 |
| DBIL | 14.1 | 15.8 |
| IDBL | 30.3 | 40.5 |
| Color ultrasound | negative | negative |

Note : WBC : White blood cells(×10^9^/L ), reference range : 3.85~10; RBC : red blood cells (×10^12^/L ), reference range : 3.1~4.5 ; HB : Hemoglobin ( g/L ), reference range : 110~149 ; PLT : platelet (×10^9^/L ), reference range : 100~320 ; HCT : hematocrit ( % ), reference range : 35~45 ; MCV : mean corpuscular volume ( fl ), reference range : 80~98 ; MCH : mean hemoglobin ( pg ), reference range : 25~35 ; MCHC : mean hemoglobin concentration ( g/L ), reference range : 300~360 ; RDW-SD : red blood cell distribution width ( fL ), reference range : 32.3~42.4 ; RDW-CV ( % ) ; RET # : absolute value of reticulocyte (×10^9^/L ), reference range : 25~75 ; RET : reticulocyte percentage ( % ) ; TBIL : total bilirubin (μmol/ L ), reference range : 2~22 ; DBIL : Direct bilirubin (μmol/L ), reference range : 1~8 ; IDBL : Indirect bilirubin (μmol/L ), reference range : 1~19 .
